# Increased occurrence of PTSD symptoms in adolescents with major depressive disorder soon after the start of the COVID-19 outbreak in China: a cross-sectional survey

**DOI:** 10.1186/s12888-021-03400-1

**Published:** 2021-08-09

**Authors:** Hang Zhang, Hanmei Xu, Lijuan Huang, Yanping Wang, Fang Deng, Xiaolan Wang, Xiaowei Tang, Wo Wang, Xia Fu, Yuanmei Tao, Li Yin

**Affiliations:** 1 Mental Health Center, West China Hospital, Sichuan University, No. 28 Dianxin South Street, Sichuan 610041 Chengdu, China; 2 The Fourth People’s Hospital of Chengdu, Chengdu, 610036 Sichuan China; 3 University-Town Hospital of Chongqing Medical University, 55 University Town Middle Road, Shapingba District, Chongqing, 400000 China; 4 Institute for System Genetics, Frontiers Science Center for Disease-related Molecular Network, Chengdu, 610041 Sichuan China

**Keywords:** COVID-19, PTSD, MDD, Adolescents, CRIES-13

## Abstract

**Background:**

The Coronavirus Disease 2019 (COVID-19) pandemic continues to threaten the physical and mental health of people across the world. This study aimed to understand the psychological impact of this disease on adolescents with major depressive disorder (MDD) at 1 month after the start of the outbreak in China.

**Methods:**

Using the Children’s Impact of Event Scale (CRIES-13) questionnaire, we investigated the occurrence of posttraumatic stress disorder (PTSD) in two groups of adolescents: MDD patients who were in continuous antidepressant therapy and healthy controls. Total scores and factor subscores were compared between the two groups and subgroups stratified by sex and school grade. Logistic regression was used to identify variables associated with high total CRIES-13 scores.

**Results:**

Compared to controls (*n* = 107), the MDD group (*n* = 90) had higher total CRIES-13 scores and a higher proportion with a total score ≥ 30. They also had a lower intrusion subscore and a higher arousal subscore. In the MDD group, males and females did not differ significantly in total CRIES-13 scores or factor subscores, but junior high school students had higher avoidance subscores than senior high school students. Logistic regression showed high total CRIES-13 scores to be associated with MDD and the experience of “flashbacks” or avoidance of traumatic memories associated with COVID-19.

**Conclusions:**

It is crucial to understand the psychological impact of COVID-19 on adolescents with MDD in China, especially females and junior high school students. Long-term monitoring of adolescents with a history of mental illness is required to further understand these impacts.

**Trial registration:**

ChiCTR, ChiCTR2000033402, Registered 31 May 2020,

**Supplementary Information:**

The online version contains supplementary material available at 10.1186/s12888-021-03400-1.

## Background

Over the last several months, the COVID-19 pandemic has become the source of enormous stress for people across the world [1–4]. Studies show that individuals who experience such stressful events are more likely to suffer from depression and experience symptoms of posttraumatic stress disorder (PTSD) [5–7]. Compared to adults, adolescents are at higher risk of mental illness after disasters [8]. Therefore, it is important not to overlook the mental health of children and adolescents [9], especially because it could lead to an increased risk of psychiatric disease in adulthood [10].

Two weeks after COVID-19 breaking out in China, about 40% Chinese adolescents were found to be prone to serious psychological problems and approximately 14.4% of them experienced PTSD symptoms [11]. Younger children were found more sensitive than older adolescents in mood changes over time [12]. The moderating factors such as gender, social connection, family conflicts and media exposure were considered to affect the mental health of adolescents during this period [13]. Having prior psychiatric diagnoses especially major depressive disorder (MDD) predicted a higher vulnerability to the posttraumatic stress symptoms (PTSS) in COVID-19 pandemic [14, 15]. Being female, social disconnection, low education level and excessive exposure to media were the risk factors of high stress symptoms [11, 16]. While family and social support can help the adolescents promoting resilience during the COVID-19 crisis [13, 17].

Pre-trauma mental distress were associated with an increased occurrence of PTSD symptoms in the young adults aged 18–21 years old [18]. However, very few cross-sectional studies have addressed the association between pre-trauma mental illness and PTSD in adolescents. In this study, we investigated the PTSS in MDD adolescents after COVID-19 outbreak in China. We aim to verify the specific impact of MDD on PTSS and no other cross-sectional studies as we known were on the occurrence of PTSD symptoms in MDD adolescents during the COVID-19 outbreak. And we suspected that MDD was a risk factor of PTSS in COVID-19 pandemic.

## Methods

### Study subjects

The results described here were obtained within the context of a larger, prospective cohort study. The study was approved by the Ethics Committee of the West China Hospital of Sichuan University, and registered in the Chinese Clinical Trail Registry (ChiCTR2000033402). Written informed consent was obtained from all participants and their guardians.

Adolescents aged 12–18 years and diagnosed independently by two senior psychiatrists at our hospital with drug naïve, first-episode MDD according to *Diagnostic and Statistical Manual of Mental Disorders IV* (DSM-IV) were consecutively recruited from June 2019 to the time point we did this survey (February 2020). All adolescents are right-handed, primary school education or above, judged by imaging physician that there is no structural abnormality of the brain, can understand the content of the scale, has not received electrical convulsive therapy, has no physical illness and has not taken any other drugs recently. Before the administration of continuous antidepressant therapy, we evaluated the severity of depression symptoms using the Chinese version of Beck Depression Inventory-II (BDI-II) [19]. Age- and gender-matched junior and senior school students in Sichuan Province without COVID-19 (based on our self-report general information form) were enrolled as healthy controls. They were chosen from our another large-scale research about the psychological impact of COVID-19 on adolescents. We excluded all the participants with a history of substance abuse, as well as those suffering from severe physical and mental illnesses, including Axis I and Axis II disorders based on the DSM-IV. Nearly all subjects came from Sichuan province in China.

Based on the data that PTSD prevalence is 14.4% in Chinese youth after the outbreak of COVID-19 and 36% of depressed patients were screened out as PTSD [11, 20], we calculated the minimal sample size by Power Analysis and Sample Size version 11.0 software (PASS 11. NCSS, LLC. Kaysville, Utah, USA). We should include at least 61 MDD patients and 61 healthy controls with the power 0.8, type I error 0.05. We increased this by 10% to 68 to compensate for missing or uncooperative participants. Ultimately, we indeed recruited 95 patients and 120 controls in our research.

### Data collection

Between 17 February and 23 February, approximately 1 month after the outbreak of COVID-19 in China, we collected demographic data using a general information form that we developed which including age, gender, grade, family structure, occupation of parents, exposure to infection, relatives with COVID-19 (yes/no), having family members who work at the frontline in medical facilities (yes/no), etc. The BDI-II and the Chinese version of the Children’s Revised Impact of Event Scale (CRIES-13) were used to evaluated the severity of depressive symptoms and PTSS respectively [21].

The CRIES-13 was used as a brief self-rating scale for children ≥8 years old. The degree of each stressful symptoms in the last 2 weeks was recorded to 4 grades (not at all, rarely, sometimes, often), respectively scored 0, 1, 3, 5 points. There was no reverse entry, and the three factors were intrusion (item 1,4,8,9), avoidance (item 2,6,7,10), and high arousal (item 3, 5, 11, 12, 13), respectively corresponding to the three symptom groups of re-experience, avoidance, and high arousal in the DSM-IV diagnostic criteria for PTSD. The total CRIES-13 score ranged 0–65 points and a total score ≥ 30 was set as a cut-off point. Individuals being diagnosed with PTSD or whose score met cut-off point were considered to have significant PTSS. According to the preliminary investigation, the Cronbach number of CRIES-13 was 0.81, the re-test reliability was 0.79, and the three-factor structure was stable, which confirmed that the table was a good screening tool for assessing the degree of stress response after traumatic events experienced by children [22–24]. All the questionnaires were distributed via WeChat ensured the responses of participants were anonymous to others but can only be seen by the researchers. Additionally, we conducted telephone interviews and semi-structured mental health examinations based on the Kiddie Schedule for Affective Disorders and Schizophrenia-Present and Lifetime Version (KSADS-PL) [25]. All PTSD diagnoses were confirmed by two independent psychiatrists based on the DSM-IV*.*

### Statistical analysis

Statistical analyses were performed using Statistical Product and Service Solutions (SPSS) 25.0 (IBM, Armonk, NY, USA), and the significance level was set at *α* = 0.05. We analyzed participant data and compared CRIES-13 scores across groups using chi-squared, Mann-Whitney U, Kruskal-Wallis H, Student’s *t* or Fisher’s exact tests as appropriate.

Bivariate logistic regression analysis was performed to assess the factors influencing the proportion of subjects with a total CRIES-13 score ≥ 30. Participants were stratified into two groups: those with a total score < 30 (CRIES-) and those with a total score ≥ 30 (CRIES+). We considered the effects of group (MDD/control), sex (female/male), grade (senior/junior high school), and family structure (with single-parent family serving as reference in the regression). We also considered exposure to infection, which was defined as relatives with COVID-19 (yes/no), or as having family members who work at the frontline in medical facilities (yes/no). We included participants’ responses to such questions as “How often do images associated with COVID-19 cross your mind?” and “How often do you have to stop yourself from thinking about COVID-19?” (those responding “not at all” served as reference in the regression). Finally, we did Pearson correlation analysis between the the BDI and CRIES-13 scores in the MDD group.

## Results

### Demographic characteristics

Of the 95 patients and 120 controls initially enrolled, valid questionnaires were received from 90 patients and 107 controls (excluded controls who had undergone psychological counseling with underlying psychological problems and MDD patients who had not been treated). Most of the patients were receiving antidepressant medications at the start of the study, including sertraline (32 patients, 50–150 mg/day), fluoxetine (7 patients, 20-40 mg/day), escitalopram (20 patients, 10-20 mg/day), venlafaxine (22 patients, 150-225 mg/day), agomelatine (4 patients, 25-50 mg/day), and one patients were taking both sertraline (50 mg/day) and agomelatine (25 mg/day). Four patients were receiving psychotherapy instead of antidepressant medication.

The MDD group (72 females) consisted of 47 junior high school students and 43 senior high school students, while the control group (82 females) had 58 junior high school students and 49 senior high school students. Patients and controls differed significantly in family structure and occupation of parents, but not in sex ratio, age, school grade or exposure to infection (Table 1).

**Table 1:** Demographic characteristics of adolescents with or without major depressive disorder, stratified by sex and school grade

Characteristic	Total				Stratified by sex						Stratified by school grade					
					Male (***n*** = 43)		Female (***n*** = 154)				Junior (***n*** = 105)		Senior (***n*** = 92)			
	MDD^**a**^	Control			MDD	Control	MDD	Control			MDD	Control	MDD	Control		
	(n = 90)	(n = 107)	z/***x***^**2**^	***p***	(n = 18)	(***n*** = 25)	(***n*** = 72)	(***n*** = 82)	H/t/***x***^**2**^	***p***	(***n*** = 47)	(***n*** = 58)	(n = 43)	(***n*** = 49)	H/t/***x***^**2**^	*p*
**Median age (years)**	15	15	−0.69	0.491	15.00	15.00	15.00	15.00	0.61	0.894	14.00	14.00	16.00	17.00	137.75	<0.001
**Baseline BDI**^**b**^ **score**	35.81 ± 11.79	NA			33.33 ± 9.11	NA	36.43 ± 12.35	NA	−1.00	0.322	37.65 ± 12.79	NA	33.80 ± 10.38	NA	1.56	0.123
**Sex (Male/Female)**	18/72	25/82	0.32	0.569							10/37	15/43	8/35	10/39	0.881	0.830
**Grade (Junior/Senior)**	47/43	58/49	0.08	0.781	10/8	15/10	37/35	43/39	0.62	0.892	NA	NA	NA	NA	NA	NA
**Family structure**			12.28	0.006						0.075						0.093
Single parent	13	8			1	2	12	6			6	4	7	4		
Two parents	34	36			6	10	28	26			17	18	17	18		
Multi-generational	19	46			5	10	14	36			9	25	10	21		
Other	24	17			6	3	18	14			15	11	9	6		
**Occupation of parents**				0.035						0.087						0.412
Medical staff	2	1			0	1	2	0			1	1	1	0		
Police	1	1			0	0	1	1			0	1	1	0		
Civil servant	8	5			0	0	8	5			4	4	4	1		
Teacher	9	3			1	1	8	2			3	1	6	2		
Freelancer	14	20			6	6	8	14			7	11	7	9		
Farmer	1	9			0	3	1	6			0	4	1	5		
Researcher	2	0			1	0	1	0			2	0	0	0		
Worker	17	24			2	6	15	18			8	12	9	12		
Self-employed	19	31			5	7	14	24			12	17	7	14		
Others	17	13			3	1	14	12			10	7	7	6		
**Respondent has family members who are**																
Infected (Yes/No)	0/90	4/103		0.127	0/18	0/25	0/72	4/78		0.249	0/47	2/56	0/43	2/47		0.342
Frontline health worker (Yes/No)	3/87	3/104		1.000	0/18	0/25	3/69	3/79		0.925	2/45	1/57	1/42	2/47		0.852

Values are n or mean ± SD, unless otherwise noted

^a^ *MDD* Major depressive disorder

^b^ *BDI* Beck Depression Inventory

The baseline mean BDI score in the MDD group (before treatment with antidepressants) was 35.81 ± 11.79. We found no significant difference in baseline BDI scores between male and female patients (*t* = − 1.00, *p* = 0.322), or between junior and senior high school students (*t* = 1.56, *p* = 0.123). They were all received antidepressant therapy at least 6 weeks and based on the BDI scores after treatments, 8 of the patients were remission on the MDD (BDI-II score ≤ 9), 12 of the patients were responders to antidepressant therapy (BDI-II score reduction rate ≥ 50%, but score > 9), 23 of the patients were partial responders to antidepressant therapy (BDI-II score reduction < 50%, but≥25%) and the other patients were actively symptomatic [26–28].

### CRIES-13 scores

The MDD group had significantly higher total CRIES-13 scores than controls (23.5 vs 21, *z* = − 2.14, *p* = 0.033), as well as a higher proportion of participants with a total score ≥ 30 (34.4% vs 15.9%, χ ^2^ = 9.13, *p* = 0.003). Patients also had a significantly lower intrusion subscore (7 vs 9, *z* = − 2.17, *p* = 0.030) and higher arousal subscore (13 vs 8, *z* = − 6.39, *p* < 0.001) (Table 2).

**Table 2:** CRIES-13 scores of adolescents with or without major depressive disorder (MDD)

	Total	MDD	Controls	***z/x***^***2***^	***p***
	(***n*** = 197)	(n = 90)	(n = 107)		
**Median scores**					
Total	22.00	23.50	21.00	−2.14	0.033
Intrusion factor	8.00	7.00	9.00	−2.17	0.030
Avoidance factor	3.00	3.50	3.00	−0.14	0.886
Arousal factor	10.00	13.00	8.00	−6.39	< 0.001
**Distribution by total score, n (%)**					
<30	149 (75.6)	59 (65.6)	90 (84.1)	9.13	0.003
≥ 30	48 (24.4)	31 (34.4)	17 (15.9)		

Male and female patients did not differ significantly in total scores, factor subscores, or proportion of participants with a total score ≥ 30 (Table 3). Among controls, however, females had higher total scores (22.5 vs 16, *z* = − 2.89, *p* = 0.004) and arousal subscores (8 vs 4, *z* = − 3.79, *p* < 0.001) than males.

**Table 3:** CRIES-13 scores of adolescents with or without major depressive disorder (MDD), stratified by sex

	MDD				Controls			
	Male	Female	***z/x***^***2***^	***p***	Male	Female	***z/x***^***2***^	***p***
	(n = 18)	(n = 72)			(n = 25)	(n = 82)		
**Median scores**								
Total	22.00	25.00	−0.09	0.928	16.00	22.50	−2.89	0.004
Intrusion factor	4.50	8.00	−0.81	0.421	7.00	9.50	−1.83	0.068
Avoidance factor	4.00	3.00	−0.69	0.488	3.00	3.50	−0.47	0.637
Arousal factor	13.00	13.00	−0.20	0.840	4.00	8.00	−3.79	< 0.001
**Distribution of total scores, n (%)**								
<30	12 (66.7)	47 (65.3)	0.01	0.912	21 (84)	69 (84.1)		1.000
≥ 30	6 (33.3)	25 (34.7)			4 (16)	13 (15.9)		

Among either patients or controls, the proportion of participants with a total score ≥ 30 was not significantly different between junior high school or senior high school students (Table 4). Among patients, junior high school students had significantly higher avoidance subscores than senior high school students (5 vs 2, *z* = − 2.20, *p* = 0.028). Among controls, senior high school students had higher total scores (24 vs 19, *z* = − 2.06, *p* = 0.040) and intrusion subscores (10 vs 8, *z* = − 2.14, *p* = 0.033) than junior high school students.

**Table 4:** CRIES-13 scores of adolescents with and without major depressive disorder (MDD), stratified by school grade

	MDD				Controls			
	Junior high	Senior high	***z/x***^***2***^	***p***	Junior high	Senior high	***z/x***^***2***^	***p***
	(n = 47)	(n = 43)			(n = 58)	(n = 49)		
**Median scores**								
Total	26.00	22.00	−0.92	0.357	19.00	24.00	−2.06	0.040
Intrusion factor	6.00	7.00	−0.02	0.981	8.00	10.00	−2.14	0.033
Avoidance factor	5.00	2.00	−2.20	0.028	3.00	3.00	−0.17	0.862
Arousal factor	13.00	13.00	−0.55	0.579	7.00	8.00	−1.95	0.052
**Distribution of total scores, n (%)**								
<30	27 (57.4)	32 (74.4)	2.87	0.091	52 (89.7)	38 (77.6)	2.91	0.088
≥ 30	20 (42.6)	11 (25.6)			6 (10.3)	11 (22.4)		

### PTSD diagnosis

Two clinicians performed mental health examinations of all the subjects who had total CRIES-13 scores ≥30 (31 patients and 17 controls). Eleven patients were determined by KSADS to have experienced PTSD before the COVID-19 outbreak, while none of the controls was diagnosed with PTSD.

### Factors influencing total CRIES-13 scores

Logistic regression identified the following factors as influencing total CRIES-13 scores: group, responses to CRIES-13 questions such as COVID-19 images cross one’s mind, and having to stop oneself from thinking about the pandemic (Table 5). The model was able to classify 85.3% of participants into correct CRIES- and CRIES+ groups, corresponding to sensitivity, 60.4%; specificity, 93.3%; positive predictive value,74.4%; and negative predictive value, 88% (Table 6). After we excluded the 11 patients experiencing PTSD previously, the results didn’t change (see Supplementary materials).

**Table 5:** Logistic regression to identify related factors to a total CRIES-13 score of at least 30 (n = 197)

Variable	B	SE	Wald chi-square	OR(95% CI)	***p***
**Group** (Patient/Control)	2.92	0.63	21.39	18.54 (5.38–63.88)	< 0.001
**Sex** (Female/Male)	0.49	0.64	0.60	1.63 (0.47–5.68)	0.441
**Grade** (Senior high/Junior high school)	0.43	0.50	0.75	1.54 (0.58–4.07)	0.386
**Family structure**^**a**^			4.64		0.200
Two parents	0.01	0.80	0.00	1.01 (0.21–4.86)	0.990
Multi-generational	1.30	0.86	2.28	3.66 (0.68–19.69)	0.131
Other	0.13	0.92	0.02	1.14 (0.19–6.86)	0.885
**Relative(s) infected with COVID-19** (Yes/No)	0.98	1.49	0.44	2.68 (0.14–49.56)	0.509
**Family member works at frontline** (Yes/No)	−0.66	1.79	0.13	0.52(0.02–17.43)	0.714
**How often do images associated with COVID-19 cross your mind?**^**b**^			27.16		< 0.001
Rarely	1.64	1.17	1.96	5.16 (0.52–51.43)	0.162
Sometimes	4.01	1.16	11.96	54.89 (5.67–531.26)	0.001
Often	5.81	1.34	18.96	333.93 (24.42–4567.19)	< 0.001
**How often do you have to stop yourself from thinking about COVID-19?**^**c**^			21.97		< 0.001
Rarely	1.66	0.65	6.60	5.24(1.48–18.57)	0.010
Sometimes	3.36	0.74	20.83	28.81(6.81–122.01)	< 0.001
Often	3.38	1.10	9.56	29.23(3.44–248.23)	0.002

^a^ Respondents answering “single-parent family” served as the reference group

^b,c^Respondents answering “not at all” for these questions served as the reference group

**Table 6:** Classification table of the logistic regression

Observed	Predicted by combination of all factors		% correct
	CRIES-13 -	CRIES-13 +	
CRIES-13 -	139	10	93.3
CRIES-13 +	19	29	60.4
**Overall**			85.3

CRIES-13 -: respondents with total CRIES-13 scores<30, CRIES-13 +: respondents with total CRIES-13 scores ≥30

### Relationship between the BDI and CRIES-13 scores in the MDD group

**Fig. 1:**
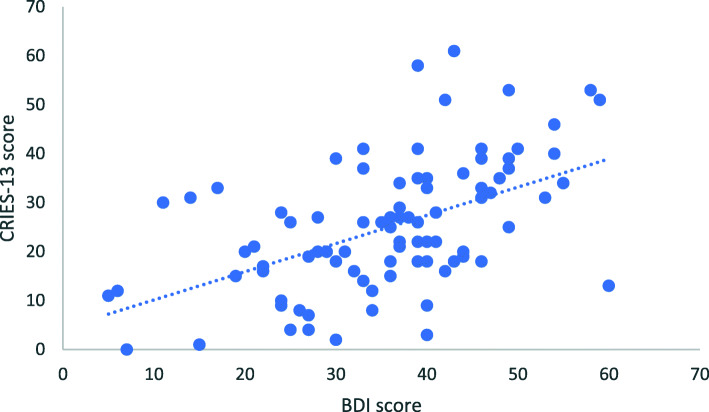
Positive correlation between the BDI and CRIES-13 total score in MDD patients (n = 90)

There was a moderate positive correlation between the BDI and CRIES-13 total scores (r = 0.511, *p* < 0.001) (Fig. 1).

## Discussion

In this study, we examined and compared the effects of the COVID-19 outbreak on the mental health of Chinese adolescents with or without MDD. We found that the MDD group had higher total CRIES-13 scores, as well as a higher proportion of participants with a total score ≥ 30 than healthy controls. These results indicate that early in the COVID-19 outbreak, adolescents with MDD were more likely than adolescents without MDD to experience severe psychological stress and symptoms of PTSD. Similar results were observed in United States military personnel deployed in the conflicts in Iraq and Afghanistan and patients with neurotic disorders after the Great East Japan Earthquake on 11 March 2011 [29, 30].

Our study found higher arousal and lower intrusion subscores among patients than controls based on the CRIES-13. The symptoms of arousal observed in the MDD group were inability to concentrate, irritability, insomnia, as well as increased vigilance and fear. This could be due to higher levels of brain arousal and hyper-stable regulation caused by the hyperactivity of the locus coeruleus in the central noradrenergic system [31, 32]. The hyper-stable regulation mode can prevent MDD patients from reducing arousal in order to sleep or regulate diurnal variation in mood [33]. The lower intrusion levels in MDD patients may be a result of antidepressant treatment, which can decrease the response to psychological stimuli by dampening activation of the amygdala [34, 35] .The amygdala is activated when the brain encodes non-spontaneous, intrusive memories, such as “flashbacks” of traumatic events [36, 37] . Further studies should examine how arousal and intrusion levels influence risk of PTSD symptoms in adolescents during the COVID-19 outbreak.

Age is a crucial factor associated with psychological stress caused by disasters [38, 39] . In our study, junior high school students with MDD showed higher avoidance levels than senior high school students. This may reflect higher “distractibility” and greater tendency to apply maladaptive coping strategies among younger students, who may therefore be more vulnerable to negative effects of stressful situations [40] . So we should focus on intervention of MDD patients in junior high students and try to explore how age factor affect the avoidance level of MDD patients.

Studies have also reported sex differences in the severity of posttraumatic stress symptoms [41, 42] . After the 5.12 Wenchuan earthquake in China, women were at higher risk of PTSD [42], and this increased risk of PTSD may be associated with biological, environmental and social factors [43] .Fluctuations in ovarian hormone levels can alter sensitivity to emotional stimuli at certain stages of the menstrual cycle, which could in turn lead to an increased susceptibility to mental illness [44]. A study on adult women showed that fear associated with the COVID-19 outbreak can lead to higher arousal symptoms [45]. Among individuals with PTSD, women show greater reactivity than men in the fear and arousal network in the ventral region of the brainstem [46] . These reports are consistent with our findings that female controls had higher total CRIES-13 scores and arousal subscores than male controls. However, we observed no significant sex differences in total scores or factor subscores among MDD patients. MDD has been associated with sensitization of the neurotransmitter and neuroendocrine systems, resulting in higher reactivity to stress [47], but whether women with MDD are more vulnerable to external stress than men with MDD is unknown. We speculate that the MDD-induced shift in hormone levels may mask sex differences in reactivity to external stimuli.

Adolescents can recover from the effects of their maladaptive cognitive style after disasters sooner than adults can [48]. Nevertheless, there is a positive correlation between history of pre-traumatic mental illness and post-traumatic stress symptoms in both adults and adolescents [48, 49], and our results with adolescents show that larger proportions of patients than controls had PTSD symptoms and total CRIES-13 scores ≥30. The higher pre-traumatic BDI scores was associated with a higher posttraumatic CRIES-13 scores. Therefore, the post-disaster mental status of MDD adolescents with high BDI scores should be carefully monitored to avoid the risk of PTSD. A hallmark of PTSD is the transition from re-experiencing to avoiding memories associated with trauma [36]. Consistent with other studies on the role of avoidance symptoms [50, 51], we observed a significant association between total CRIES-13 scores and responses to questions such as“ How often do images associated with COVID-19 cross your mind?” and “How often do you have to stop yourself from thinking about COVID-19?” The mechanisms of how these factors associated with high risk of PTSD occurrence can be studied in the future. In our study, patients and controls differed significantly in family structure and occupation of parents. We thought these different factors maybe the risk factors of MDD or PTSS. So we continued the study and included them as the independent variable in the logistic regression to identify related factors to a total CRIES-13 score of at least 30. The final results showed that they had no contributions to the CRIES-13 score.

## Conclusion

Our cross-sectional study provides what appears to be the first evidence that COVID-19 is having a particularly strong psychological impact on adolescents with MDD, especially females and junior high school students. So focusing on these adolescents in high-risk and offering mental interventions to them in time was crucial after the crisis. And our results may provide some references for future mental health problem prevention in adolescents when the similar crisis happen. Whether our findings can be generalized to adolescents outside Sichuan province in China needs to be confirmed. Future work should examine potential influence of antidepressant therapy on the vulnerability of adolescents to COVID-19 stress. Based on the mental health examinations that we conducted on a subset of patients and controls who scored at least 30 on CRIES-13, we did not detect anyone with PTSD that could be attributed to the COVID-19 outbreak. This may be because our study was conducted only 1 month after the outbreak began in China, and because of the containment measures implemented rapidly by the government. Our continuing analysis of these patients and controls as part of our larger prospective cohort study (ChiCTR2000033402) may detect an association between COVID-19 and PTSD among adolescents with or without MDD. This may help identify adolescents who require psychological and clinical intervention.

## Supplementary Information

**Additional file 1: Table S1.** Demographic characteristics of adolescents with or without major depressive disorder (excluded prior PTSD diagnosis), stratified by sex and school grade (*n*=186). **Table S2.** CRIES-13 scores of adolescents with or without major depressive disorder (MDD) (excluded prior PTSD diagnosis) (*n*=186). **Table S3.** CRIES-13 scores of adolescents with or without major depressive disorder (MDD), stratified by sex (excluded prior PTSD diagnosis) (*n*=186) . **Table S4.** CRIES-13 scores of adolescents with and without major depressive disorder (MDD), stratified by school grade  (excluded prior PTSD diagnosis) (*n*=186). **Table S5.** Logistic regression to identify related factors to a total CRIES-13 score of at least 30 (*n* = 186). **Table S6.** Classification table of the logistic regression (*n*=186). **Fig. S1.** Positive correlation between the BDI and CRIES-13 total score in MDD patients (*n* = 79).

## Data Availability

The data that support the findings of this study are available on request from the corresponding author (Li Yin, yli009@163.com). The data are not publicly available due to privacy or ethical restrictions.

## Abbreviations

BDI-II: Beck Depression Inventory-II
COVID-19: Coronavirus Disease 2019
CRIES-13: the Children’s Impact of Event Scale questionnaire
DSM-IV: the Diagnostic and Statistical Manual of Mental Disorders IV
KSADS-PL: Kiddie Schedule for Affective Disorders and Schizophrenia-Present and Lifetime Version
MDD: Major Depressive Disorder
PTSD: Post-Traumatic Stress Disorder
PTSS: Post-Traumatic Stress Symptom
SPSS: Statistical Product and Service Solutions
PASS: Power Analysis and Sample Size

## References

[1] Tang W, Hu T, Hu B, Jin C, Wang G, Xie C (2020). Prevalence and correlates of PTSD and depressive symptoms one month after the outbreak of the COVID-19 epidemic in a sample of home-quarantined Chinese university students. J Affect Disord.

[2] Liu CH, Zhang E, Wong GTF, Hyun S, Hahm HC (2020). Factors associated with depression, anxiety, and PTSD symptomatology during the COVID-19 pandemic: clinical implications for U.S. young adult mental health. Psychiatry Res.

[3] Lai J, Ma S, Wang Y, Cai Z, Hu J, Wei N, Wu J, Du H, Chen T, Li R et al: Factors Associated With Mental Health Outcomes Among Health Care Workers Exposed to Coronavirus Disease 2019. JAMA Netw Open. 2020;3(3):e203976. 10.1001/jamanetworkopen.2020.3976 PMC7090843 32202646

[4] Fawaz M, Samaha A (2020). COVID-19 quarantine: post-traumatic stress symptomatology among Lebanese citizens. Int J Soc Psychiat.

[5] Stander VA, Thomsen CJ, Highfill-McRoy RM (2014). Etiology of depression comorbidity in combat-related PTSD: a review of the literature. Clin Psychol Rev.

[6] Breslau N (2009). The epidemiology of trauma, PTSD, and other posttrauma disorders. Trauma Violence Abuse.

[7] Brady KT, Killeen TK, Brewerton T, Lucerini S (2000). Comorbidity of psychiatric disorders and posttraumatic stress disorder. J Clin Psychiatry.

[8] Kolaitis G, Kotsopoulos J, Tsiantis J, Haritaki S, Rigizou F, Zacharaki L (2003). Posttraumatic stress reactions among children following the Athens earthquake of September 1999. Eur Child Adolesc Psychiatry.

[9] Danese A, Smith P, Chitsabesan P, Dubicka B (2020). Child and adolescent mental health amidst emergencies and disasters. Br J Psychiatry.

[10] Xu W, Yuan G, Liu Z, Zhou Y, An Y (2018). Prevalence and predictors of PTSD and depression among adolescent victims of the summer 2016 tornado in Yancheng City. Arch Psychiatr Nurs.

[11] Liang L, Ren H, Cao R, Hu Y, Qin Z, Li C (2020). The effect of COVID-19 on youth mental health. Psychiatr Q.

[12] Green KH, van de Groep S, Sweijen SW, Becht AI, Buijzen M, de Leeuw RNH (2021). Mood and emotional reactivity of adolescents during the COVID-19 pandemic: short-term and long-term effects and the impact of social and socioeconomic stressors. Sci Rep.

[13] Magson NR, Freeman JYA, Rapee RM, Richardson CE, Oar EL, Fardouly J (2021). Risk and protective factors for prospective changes in adolescent mental health during the COVID-19 pandemic. J Youth Adolesc.

[14] Gonzalez-Sanguino C, Ausin B, Castellanos MA, Saiz J, Lopez-Gomez A, Ugidos C (2020). Mental health consequences during the initial stage of the 2020 coronavirus pandemic (COVID-19) in Spain. Brain Behav Immun.

[15] Di Nicola M, Dattoli L, Moccia L, Pepe M, Janiri D, Fiorillo A (2020). Serum 25-hydroxyvitamin D levels and psychological distress symptoms in patients with affective disorders during the COVID-19 pandemic. Psychoneuroendocrinology.

[16] Yue J, Zang X, Le Y, An Y. Anxiety, depression and PTSD among children and their parent during 2019 novel coronavirus disease (COVID-19) outbreak in China. Curr Psychol. 2020. p. 1–8. 10.1007/s12144-020-01191-4. Epub ahead of print. 10.1007/s12144-020-01191-4 PMC7666617 33223783

[17] Dvorsky MR, Breaux R, Becker SP. Finding ordinary magic in extraordinary times: child and adolescent resilience during the COVID-19 pandemic. Eur Child Adolesc Psychiatry. 2020. p. 1:1–3. 10.1007/s00787-020-01583-8. Epub ahead of print. 10.1007/s00787-020-01583-8 PMC7327857 32613259

[18] Frazier PA, Gavian M, Hirai R, Park C, Tennen H, Tomich P (2011). Prospective predictors of posttraumatic stress disorder symptoms: direct and mediated relations. Psychol Trauma Theory Res Pract Policy.

[19] Yang W, Liu S, Zhou T, Peng F, Liu X, Li L (2014). Reliability and validity of Chinese version of the Beck depression inventory-II in Chinese adolescents. Chin J Clin Psychol.

[20] Campbell DG, Felker BL, Liu CF, Yano EM, Kirchner JE, Chan D (2007). Prevalence of depression-PTSD comorbidity: implications for clinical practice guidelines and primary care-based interventions. J Gen Intern Med.

[21] Mingjing S, Yi H, Yi Z, Hui F, Wang D, Lushi J, Wei Z (2009). Psychometric properties of the Children's revised impact of event scale in children from earthquake affected areas. Chin J Psychiatry.

[22] Perrin S, Meiser-Stedman R, Smith P (2005). The Children's revised impact of event scale (CRIES): validity as a screening instrument for PTSD. Behav Cognit Psychther.

[23] Giannopoulou L, Smith P, Ecker C, Strouthos M, Dikaiakou A, Yule W (2006). Factor structure of the Children's revised impact of event scale (CRIES) with children exposed to earthquake. Pers Individ Differ.

[24] Smith P, Perrin S, Dyregrov A, Yule W (2003). Principal components analysis of the impact of event scale with children in war. Pers Individ Differ.

[25] Kaufman J, Birmaher B, Brent D, Rao U, Flynn C, Moreci P (1997). Schedule for affective disorders and schizophrenia for school-age children present and lifetime version (K-SADS-PL): initial reliability and validity data. J Am Acad Child Adolesc Psychiatry.

[26] Freedland KE, Carney RM, Rich MW, Steinmeyer BC, Rubin EH (2015). Cognitive behavior therapy for depression and self-Care in Heart Failure Patients: a randomized clinical trial. JAMA Intern Med.

[27] Nierenberg AA, DeCecco LM (2001). Definitions of antidepressant treatment response, remission, nonresponse, partial response, and other relevant outcomes: a focus on treatment-resistant depression. J Clin Psychiat.

[28] Streeter CC, Gerbarg PL, Whitfield TH, Owen L, Johnston J, Silveri MM (2017). Treatment of major depressive disorder with Iyengar yoga and coherent breathing: a randomized controlled dosing study. J Altern Complement Med.

[29] Sandweiss DA, Slymen DJ, Leardmann CA, Smith B, White MR, Boyko EJ (2011). Preinjury psychiatric status, injury severity, and postdeployment posttraumatic stress disorder. Arch Gen Psychiatry.

[30] Inoue K, Inoue K, Suda S, Shioda K, Kobayashi T, Kishi K (2015). Differences in vulnerability to traumatic stress among patients with psychiatric disorders: one-year follow-up study after the great East Japan earthquake. Psychiatry Clin Neurosci.

[31] Schmidt FM, Sander C, Dietz ME, Nowak C, Schröder T, Mergl R (2017). Brain arousal regulation as response predictor for antidepressant therapy in major depression. Sci Rep.

[32] Hegerl U, Wilk K, Olbrich S, Schoenknecht P, Sander C (2012). Hyperstable regulation of vigilance in patients with major depressive disorder. World J Biol Psychiatry.

[33] Schmidt FM, Pschiebl A, Sander C, Kirkby KC, Thormann J, Minkwitz J (2016). Impact of serum cytokine levels on EEG-measured arousal regulation in patients with major depressive disorder and healthy controls. Neuropsychobiology.

[34] Windischberger C, Lanzenberger R, Holik A, Spindelegger C, Stein P, Moser U (2010). Area-specific modulation of neural activation comparing escitalopram and citalopram revealed by pharm aco-fMRI: a randomized cross-over study. Neuroimage.

[35] Chen YT, Huang MW, Hung IC, Lane HY, Hou CJ (2014). Right and left amygdalae activation in patients with major depression receiving antidepressant treatm ent, as revealed by fMRI. Behav Brain Funct.

[36] Brewin CR, Dalgleish T, Joseph S (1996). A dual representation theory of posttraumatic stress disorder. Psychol Rev.

[37] Bourne C, Mackay CE, Holmes EA (2013). The neural basis of flashback formation: the impact of viewing trauma. Psychol Med.

[38] Morris MC, Kouros CD, Hellman N, Rao U, Garber J (2014). Two prospective studies of changes in stress generation across depressive episodes in adolescents and emerging adults. Dev Psychopathol.

[39] Norris FH, Friedman MJ, Watson PJ, Byrne CM, Diaz E, Kaniasty K (2002). 60,000 disaster victims speak: part I. An empirical review of the empirical literature, 1981-2001. Psychiatry.

[40] Hampel P, Petermann F (2005). Age and gender effects on coping in children and adolescents. J Youth Adolescence.

[41] Alisic E, Jongmans MJ, van Wesel F, Kleber RJ (2011). Building child trauma theory from longitudinal studies: a meta-analysis. Clin Psychol Rev.

[42] Ma X, Liu X, Hu X, Qiu C, Wang Y, Huang Y (2011). Risk indicators for post-traumatic stress disorder in adolescents exposed to the 5.12 Wenchuan earthq uake in China. Psychiatry Res.

[43] McLean CP, Anderson ER (2009). Brave men and timid women? A review of the gender differences in fear and anxiety. Clin Psychol Rev.

[44] Soni M, Curran VH, Kamboj SK (2013). Identification of a narrow post-ovulatory window of vulnerability to distressing involuntary memories in healthy women. Neurobiol Learn Mem.

[45] Liu N, Zhang F, Wei C, Jia Y, Shang Z, Sun L, et al. Prevalence and predictors of PTSS during COVID-19 outbreak in China hardest-hit areas: gender differences matter. Psychiatry Res. 2020;287. 10.1016/j.psychres.2020.112921 PMC7102622 32240896

[46] Felmingham K, Williams LM, Kemp AH, Liddell B, Falconer E, Peduto A (2010). Neural responses to masked fear faces: sex differences and trauma exposure in posttraumatic stress di sorder. J Abnorm Psychol.

[47] Essau CA, Lewinsohn PM, Seeley JR, Sasagawa S (2010). Gender differences in the developmental course of depression. J Affect Disord.

[48] Felton JW, Cole DA, Martin NC (2013). Effects of rumination on child and adolescent depressive reactions to a natural disaster: the 2010 Nashville flood. J Abnorm Psychol.

[49] Sullivan G, Vasterling JJ, Han X, Tharp AT, Davis T, Deitch EA (2013). Preexisting mental illness and risk for developing a new disorder after hurricane Katrina. J Nerv Ment Dis.

[50] Giannopoulou I, Strouthos M, Smith P, Dikaiakou A, Galanopoulou V, Yule W (2006). Post-traumatic stress reactions of children and adolescents exposed to the Athens 1999 earthquake. Eur Psychiat.

[51] Sahin NH, Batigun AD, Yilmaz B (2007). Psychological symptoms of Turkish children and adolescents after the 1999 earthquake: exposure, gender, location, and time duration. J Trauma Stress.

