# Supplementary material for: Increased occurrence of PTSD symptoms in adolescents with major depressive disorder soon after the start of the COVID-19 outbreak in China: a cross-sectional survey
Source: BMC Psychiatry. 2021 Aug 9;21:395. doi: 10.1186/s12888-021-03400-1 (PMC8352150; doi:10.1186/s12888-021-03400-1)
Supplement: Supplementary file 1 — Additional file 1: Table S1. Demographic characteristics of adolescents with or without major depressive disorder (excluded prior PTSD diagnosis), stratified by sex and school grade (n=186). Table S2. CRIES-13 scores of adolescents with or without major depressive disorder (MDD) (excluded prior PTSD diagnosis) (n=186). Table S3. CRIES-13 scores of adolescents with or without major depressive disorder (MDD), stratified by sex (excluded prior PTSD diagnosis) (n=186) . Table S4. CRIES-13 scores of adolescents with and without major depressive disorder (MDD), stratified by school grade (excluded prior PTSD diagnosis) (n=186). Table S5. Logistic regression to identify related factors to a total CRIES-13 score of at least 30 (n = 186). Table S6. Classification table of the logistic regression (n=186). Fig. S1. Positive correlation between the BDI and CRIES-13 total score in MDD patients (n = 79). [file 12888_2021_3400_MOESM1_ESM.docx]

**Table S1: Demographic characteristics of adolescents with or without major depressive disorder, stratified by sex and school grade**

|  | **Total** | |  |  | **Stratified by sex** | | | |  |  | **Stratified by school grade** | | | |  |  |
| --- | --- | --- | --- | --- | --- | --- | --- | --- | --- | --- | --- | --- | --- | --- | --- | --- |
|  |  |  |  |  | **Male (n = 42)** | | **Female (n = 144)** | |  |  | **Junior (n = 98)** | | **Senior (n = 88)** | |  |  |
| **Characteristic** | **MDD^1^** | **Control** |  |  | **MDD** | **Control** | **MDD** | **Control** |  |  | **MDD** | **Control** | **MDD** | **Control** |  |  |
|  | **(n = 79)** | **(n = 107)** | **z/*x*^2^** | ***p*** | **(n = 17)** | **(n = 25)** | **(n = 62)** | **(n = 82)** | **H/t/*x*^2^** | ***p*** | **(n = 40)** | **(n = 58)** | **(n = 39)** | **(n = 49)** | **H/t/*x*^2^** | *p* |
| **Median age (years)** | 15 | 15 | -0.64 | 0.524 | 15.00 | 15.00 | 15.00 | 15.00 | 0.56 | 0.906 | 14.00 | 14.00 | 16.00 | 17.00 | 132.00 | ＜0.001 |
| **Baseline BDI^2^ score** | 34.65 ± 11.29 | NA |  |  | 33.35 ± 9.39 | NA | 35.00 ± 11.80 | NA | -0.53 | 0.596 | 36.57±12.22 | NA | 32.67±10.01 | NA | 1.55 | 0.126 |
| **Sex (Male/Female)** | 17/62 | 25/82 | 0.09 | 0.766 |  |  |  |  |  |  | 9/31 | 15/43 | 8/31 | 10/39 | 0.59 | 0.900 |
| **Grade (Junior/Senior)** | 40/39 | 58/49 | 0.23 | 0.630 | 9/8 | 15/10 | 31/31 | 43/39 | 0.72 | 0.869 | NA | NA | NA | NA | NA | NA |
| **Family structure** |  |  | 10.06 | 0.018 |  |  |  |  |  | 0.164 |  |  |  |  |  | 0.200 |
| Single parent | 10 | 8 |  |  | 1 | 2 | 9 | 6 |  |  | 5 | 4 | 5 | 4 |  |  |
| Two parents | 32 | 36 |  |  | 6 | 10 | 26 | 26 |  |  | 17 | 18 | 15 | 18 |  |  |
| Multi-generational | 17 | 46 |  |  | 5 | 10 | 12 | 36 |  |  | 7 | 25 | 10 | 21 |  |  |
| Other | 20 | 17 |  |  | 5 | 3 | 15 | 14 |  |  | 11 | 11 | 9 | 6 |  |  |
| **Occupation of parents** |  |  |  | 0.031 |  |  |  |  |  | 0.046 |  |  |  |  |  | 0.505 |
| Medical staff | 2 | 1 |  |  | 0 | 1 | 2 | 0 |  |  | 1 | 1 | 1 | 0 |  |  |
| Police | 0 | 1 |  |  | 0 | 0 | 0 | 1 |  |  | 0 | 1 | 0 | 0 |  |  |
| Civil servant | 8 | 5 |  |  | 0 | 0 | 8 | 5 |  |  | 4 | 4 | 4 | 1 |  |  |
| Teacher | 7 | 3 |  |  | 1 | 1 | 6 | 2 |  |  | 3 | 1 | 4 | 2 |  |  |
| Freelancer | 12 | 20 |  |  | 6 | 6 | 6 | 14 |  |  | 6 | 11 | 6 | 9 |  |  |
| Farmer | 1 | 9 |  |  | 0 | 3 | 1 | 6 |  |  | 0 | 4 | 1 | 5 |  |  |
| Researcher | 2 | 0 |  |  | 1 | 0 | 1 | 0 |  |  | 2 | 0 | 0 | 0 |  |  |
| Worker | 15 | 24 |  |  | 2 | 6 | 13 | 18 |  |  | 6 | 12 | 9 | 12 |  |  |
| Self-employed | 16 | 31 |  |  | 5 | 7 | 11 | 24 |  |  | 9 | 17 | 7 | 14 |  |  |
| Others | 16 | 13 |  |  | 2 | 1 | 14 | 12 |  |  | 9 | 7 | 7 | 6 |  |  |
| **Respondent has family members who are** | | |  |  |  |  |  |  |  |  |  |  |  |  |  |  |
| Infected (Yes/No) | 0/79 | 4/103 |  | 0.138 | 0/17 | 0/25 | 0/62 | 4/78 |  | 0.287 | 0/40 | 2/56 | 0/39 | 2/47 |  | 0.521 |
| Frontline health worker (Yes/No) | 3/76 | 3/104 |  | 0.700 | 0/17 | 0/25 | 3/59 | 3/79 |  | 0.921 | 2/38 | 1/57 | 1/38 | 2/47 |  | 0.807 |

Values are n or mean ± SD, unless otherwise noted.

^1^ MDD, major depressive disorder

^2^ BDI, Beck Depression Inventory

**Table S2: CRIES-13 scores of adolescents with or without major depressive disorder (MDD)**

|  | **Total** | **MDD** | **Controls** | ***z/x^2^*** | ***p*** |
| --- | --- | --- | --- | --- | --- |
|  | **(n = 186)** | **(n = 79)** | **(n = 107)** |  |  |
| **Median scores** | |  |  |  |  |
| Total | 21.00 | 21.00 | 21.00 | -0.78 | 0.434 |
| Intrusion factor | 8.00 | 6.00 | 9.00 | -3.25 | 0.001 |
| Avoidance factor | 3.00 | 2.00 | 3.00 | -1.06 | 0.290 |
| Arousal factor | 9.00 | 12.00 | 8.00 | -5.40 | <0.001 |
| **Distribution by total score, n (%)** | | | | |  |
| ＜30 | 149 (80.1) | 59 (74.7) | 90 (84.1) | 2.54 | 0.111 |
| ≥ 30 | 37 (19.9) | 20 (25.3) | 17 (15.9) |  |  |

**Table S3: CRIES-13 scores of adolescents with or without major depressive disorder (MDD), stratified by sex**

|  | **MDD** | |  |  | **Controls** | |  |  |
| --- | --- | --- | --- | --- | --- | --- | --- | --- |
|  | **Male** | **Female** | ***z/x^2^*** | ***p*** | **Male** | **Female** | ***z/x^2^*** | ***p*** |
|  | **(n = 17)** | **(n = 62)** |  |  | **(n = 25)** | **(n = 82)** |  |  |
| **Median scores** |  |  |  |  |  |  |  |  |
| Total | 22.00 | 20.00 | -0.59 | 0.554 | 16.00 | 22.50 | -2.89 | 0.004 |
| Intrusion factor | 4.00 | 6.00 | -0.56 | 0.577 | 7.00 | 9.50 | -1.83 | 0.068 |
| Avoidance factor | 4.00 | 2.00 | -1.21 | 0.228 | 3.00 | 3.50 | -0.47 | 0.637 |
| Arousal factor | 12.00 | 12.50 | -0.40 | 0.693 | 4.00 | 8.00 | -3.79 | <0.001 |
| **Distribution of total scores, n (%)** | | | | | |  |  |  |
| ＜30 | 12 (70.6) | 47 (75.8) |  | 0.755 | 21 (84.0) | 69 (84.1) |  | 1.000 |
| ≥ 30 | 5 (29.4) | 15 (24.2) |  |  | 4 (16.0) | 13 (15.9) |  |  |

**Table S4:** **CRIES-13 scores of adolescents with and without major depressive disorder (MDD), stratified by school grade**

|  | **MDD** | |  |  | **Controls** | |  |  |
| --- | --- | --- | --- | --- | --- | --- | --- | --- |
|  | **Junior high** | **Senior high** |  |  | **Junior high** | **Senior high** |  |  |
|  | **(n = 40)** | **(n = 39)** | ***z/x^2^*** | ***p*** | **(n = 58)** | **(n = 49)** | ***z/x^2^*** | ***p*** |
| **Median scores** |  |  |  |  |  |  |  |  |
| Total | 21.50 | 20.00 | -0.48 | 0.634 | 19.00 | 24.00 | -2.06 | 0.040 |
| Intrusion factor | 5.00 | 7.00 | -0.56 | 0.578 | 8.00 | 10.00 | -2.14 | 0.033 |
| Avoidance factor | 4.00 | 1.00 | -2.04 | 0.041 | 3.00 | 3.00 | -0.17 | 0.862 |
| Arousal factor | 12.00 | 13.00 | -0.088 | 0.930 | 7.00 | 8.00 | -1.95 | 0.052 |
| **Distribution of total scores, n (%)** | | | | | |  |  |  |
| ＜30 | 27 (67.5) | 32 (82.1) | 2.21 | 0.137 | 52 (89.7) | 38 (77.6) | 2.91 | 0.088 |
| ≥ 30 | 13 (32.5) | 7 (17.9) |  |  | 6 (10.3) | 11 (22.4) |  |  |

**Table S5**: **Logistic regression to identify related factors to a total CRIES-13 score of at least 30 (n = 186)**

| **Variable** | **B** | | **SE** | **Wald chi-square** | | | **OR（95% CI）** | | ***p*** |
| --- | --- | --- | --- | --- | --- | --- | --- | --- | --- |
| **Group** (Patient/Control） | 2.43 | | 0.66 | | 13.67 | 11.39 (3.14-41.36) | | <0.001 | |
| **Sex** (Female/Male） | 0.30 | | 0.65 | | 0.21 | 1.35 (0.38-4.81) | | 0.643 | |
| **Grade** (Senior high/Junior high school） | 0.56 | | 0.52 | | 1.14 | 1.74 (0.63-4.84) | | 0.285 | |
| **Family structure^1^** |  | |  | | 3.65 |  | | 0.302 | |
| Two parents | 0.26 | | 0.88 | | 0.091 | 1.30 (0.23-7.24) | | 0.763 | |
| Multi-generational | 1.33 | | 0.93 | | 2.04 | 3.79(0.61-23.61) | | 0.154 | |
| Other | 0.11 | | 1.03 | | 0.012 | 1.12 (0.15-8.39) | | 0.913 | |
| **Relative(s) infected with COVID-19** (Yes/No) | 1.15 | | 1.48 | | 0.61 | 3.16 (0.18-56.97） | | 0.436 | |
| **Family member works at frontline** (Yes/No) | -0.36 | | 1.72 | | 0.043 | 0.70 (0.02-20.20） | | 0.835 | |
| **How often do images associated with COVID-19 cross your mind?^2^** | |  |  | | 21.87 |  | | <0.001 | |
| Rarely | 1.21 | | 1.21 | | 1.00 | 3.37 (0.31-36.16) | | 0.317 | |
| Sometimes | 3.62 | | 1.17 | | 9.60 | 37.30 (3.78-368.15) | | 0.002 | |
| Often | 5.25 | | 1.36 | | 14.80 | 190.27 (13.12-2759.12) | | <0.001 | |
| **How often do you have to stop yourself from thinking about COVID-19?^3^** | | |  | | 19.93 |  | | <0.001 | |
| Rarely | 1.65 | | 0.70 | | 5.62 | 5.22 (1.33-20.44) | | 0.018 | |
| Sometimes | 3.30 | | 0.78 | | 18.04 | 27.07 (5.91-124.01) | | <0.001 | |
| Often | 3.54 | | 1.12 | | 10.04 | 34.33 (3.85-305.82) | | 0.002 | |

^1^ Respondents answering “single-parent family” served as the reference group

^2,3^Respondents answering “not at all” for these questions served as the reference group.

**Table S6: Classification table of the logistic regression**

| **Observed** | **Predicted by combination of all factors** | | **% correct** |
| --- | --- | --- | --- |
|  | CRIES-13 - | CRIES-13 + |  |
| CRIES-13 - | 140 | 9 | 94.0 |
| CRIES-13 + | 18 | 19 | 51.4 |
| **Overall** |  |  | 85.5 |

CRIES-13 -: respondents with total CRIES-13 scores＜30, CRIES-13 +: respondents with total CRIES-13 scores ≥30

**Figure S1: Positive correlation between the BDI and CRIES-13 total score in MDD patients (n=79)**
